# Botnet Detection and Mitigation Model for IoT Networks Using Federated Learning

**DOI:** 10.3390/s23146305

**Published:** 2023-07-11

**Authors:** Francisco Lopes de Caldas Filho, Samuel Carlos Meneses Soares, Elder Oroski, Robson de Oliveira Albuquerque, Rafael Zerbini Alves da Mata, Fábio Lúcio Lopes de Mendonça, Rafael Timóteo de Sousa Júnior

**Affiliations:** 1 Electrical Engineering Department (ENE), Technology College, University of Brasília (UnB), Brasília 70910-900, Brazil; samuel.soares@uiot.org (S.C.M.S.); robson@redes.unb.br (R.d.O.A.); rafael.zerbini@uiot.org (R.Z.A.d.M.); fabio.mendonca@redes.unb.br (F.L.L.d.M.); desousa@unb.br (R.T.d.S.J.); 2 Electrical Engineering Department (DAELT), Federal University of Technology—Paraná (UTFPR), Curitiba 80230-901, Brazil; oroski@utfpr.edu.br

**Keywords:** deep learning, federated learning, fog computing, DDoS, HIDPS, NIDS

## Abstract

The Internet of Things (IoT) introduces significant security vulnerabilities, raising concerns about cyber-attacks. Attackers exploit these vulnerabilities to launch distributed denial-of-service (DDoS) attacks, compromising availability and causing financial damage to digital infrastructure. This study focuses on mitigating DDoS attacks in corporate local networks by developing a model that operates closer to the attack source. The model utilizes Host Intrusion Detection Systems (HIDS) to identify anomalous behaviors in IoT devices and employs network-based intrusion detection approaches through a Network Intrusion Detection System (NIDS) for comprehensive attack identification. Additionally, a Host Intrusion Detection and Prevention System (HIDPS) is implemented in a fog computing infrastructure for real-time and precise attack detection. The proposed model integrates NIDS with federated learning, allowing devices to locally analyze their data and contribute to the detection of anomalous traffic. The distributed architecture enhances security by preventing volumetric attack traffic from reaching internet service providers and destination servers. This research contributes to the advancement of cybersecurity in local network environments and strengthens the protection of IoT networks against malicious traffic. This work highlights the efficiency of using a federated training and detection procedure through deep learning to minimize the impact of a single point of failure (SPOF) and reduce the workload of each device, thus achieving accuracy of 89.753% during detection and increasing privacy issues in a decentralized IoT infrastructure with a near-real-time detection and mitigation system.

## 1. Introduction

The growth of IoT devices has brought many benefits to industrial, rural and home environments. These devices have increased computational capacity, allowing for more advanced applications and functionalities [1]. However, this also means that there is a greater need for security measures to prevent these devices from being exploited because of attack vulnerabilities, as seen in [2], and used in botnets. Botnets are a set of devices that carry out orchestrated attacks on servers and network services, intending to cause unavailability [3]. They are formed by infected endpoints such as computers, wireless routers, mobile phones and Internet of Things devices. The latter, in turn, are built with a focus on ease of use, without being concerned with ensuring safe development. IoT devices, when infected, usually serve to attack government services, banks and large companies [4].

Intrusion Detection Systems (IDS) use various methods to detect intrusions. One method is signature-based detection, which involves looking for known intrusions in the form of signatures, patterns or rules. This method is only effective against known attacks and cannot detect unknown or zero-day attacks. Zero-day attacks exploit new vulnerabilities or old vulnerabilities in a different way, making identification by signatures ineffective for detection [5]. Another method is anomaly-based detection, which involves detecting abnormal behavior by comparing it with expected or normal behavior. This method can detect a wide range of malicious intrusions; however, it also has limitations such as the potential for false positives. Anomaly-based techniques can be divided into statistical methods, machine learning methods and other techniques based on data mining and game theory models.

Host-Based Intrusion Detection Systems (HIDS) are security systems that run on the devices themselves and can detect and correct problems in the operating system. These systems can be used on IoT devices to identify and mitigate failures in the operating system and prevent them from being exploited and used in botnets [6]. However, despite the increased computational capacity of IoT devices, they still have limitations compared to servers and network devices, which may limit their ability to run certain security solutions, such as Network Intrusion Detection Systems (NIDS).

Network Intrusion Detection Systems (NIDS) can analyze traffic from all hosts on a network and detect abnormal behavior, such as excessive attempts at connections to a server. These systems can generate alerts or perform attack mitigation, behaving similarly to Network Intrusion Prevention Systems (NIPS). NIDS software such as Suricata and Snort can process a large volume of data and generate alerts or activate external security systems. They can also send events to Big Data platforms for further analysis to identify patterns of attacks that could not be identified in real time [7].

Different machine learning (ML) and deep learning (DL) techniques have been employed to identify attacks, employing supervised and unsupervised learning. Deep learning algorithms can be trained to perceive different types of attacks and trigger security elements such as firewalls or SDN controllers to block anomalous data flows [8].

Additionally, an emerging approach in the field of cybersecurity is federated learning. This technology enables the training of machine learning models on distributed devices, such as IoT devices, without the need to share raw data among devices or send them to a central server. This approach preserves data privacy and allows IoT devices to contribute to the learning process by aggregating local knowledge to enhance the performance of the global model. In the context of attack identification, federated learning can be applied to train intrusion detection models on IoT devices, enabling them to learn from their own data and share only model updates with the central server. This not only improves the efficiency and scalability of the training process but also preserves the privacy of sensitive data. Thus, federated learning represents a promising approach to enhance cybersecurity in distributed systems, such as the IoT.

To identify anomalous traffic, the model proposes an analysis of the network data flow using an approach known as federated learning. Instead of sending the data to a central server, the model is sent to local devices, where they are trained individually. Then, only the updated model parameters are sent back to the central server to aggregate the information and generate a global model. This process enables the analysis of anomalous traffic to be performed locally on the devices, preserving data privacy and reducing the dependence on a continuous connection to a central server. By employing federated learning, the proposed model can identify suspicious traffic patterns and contribute to the detection of denial-of-service attacks in the network.

Upon identifying anomalous data flows that need to be blocked, the federated learning server makes an API request to a fog computing orchestrator, responsible for distributing the rules to IoT devices and ensuring that they block the traffic at its source. In addition to rules obtained through the federated learning framework, the fog computing orchestrator can receive signature rules to block not only anomalous traffic but also processes and any other parameters that characterize an IoT device as part of a botnet.

We can list the main contributions of this model as follows.

- The main contribution of this model is the construction of a fog computing architecture, in which end devices perform security-focused tasks determined by the orchestrator. This work has created a real environment with IoT devices to validate the proposed model.
- The definition of a set of tools capable of converting network data flows into data that can be processed using federated learning is another significant contribution of this model.
- The evolution of the proposed model of communication between HIDS systems and the orchestrator, presented in [9], is noteworthy. In this model, the orchestrator receives updates not only from an administrator but also from the federated learning server after identifying data flows that need to be blocked. This enhanced communication framework improves the overall effectiveness and responsiveness of the system in detecting and mitigating security threats.

To carry out the validation of the proposed model, we created a scenario with a real botnet, in addition to all the security components proposed in this work. In this way, it was possible, in addition to validating the proposed model, to obtain accurate measurements of the reaction times of the safety components.

Furthermore, the proposed model is not restricted to conventional networks but can be applied to Software-Defined Networks (SDNs), enabling network reconfiguration to proactively mitigate anomalous traffic closer to its origin. This capability enhances the system’s efficacy in detecting and responding to attacks, thereby bolstering the overall network security.

Besides this Introduction, this paper is organized as follows. In Section 1.1, we present the background regarding the technologies and methodologies used in the paper. In Section 2, we present a literature overview of related works. In Section 3, we present the structural view of the proposed solution and its behavior. In Section 4, we present the testing methodology used to implement the proposed solution. In Section 5, we present the results obtained from the testing methodology. Finally, in Section 6, we present our conclusions and suggestions for future work.

### 1.1. Background

This section presents and discusses academic and industry concepts and views focused on the security of the IoT domain. This work presents a diversity of topics related to IDS and IPS: botnets [10], HIDS [11], NIDS [12]. However, the most important topics that need to be specified and detailed are described thereafter.

#### 1.1.1. Intrusion Response System

Intrusion Response Systems (IRS), which represent a concept that is starting to be used in intrusion detection works, such as [13], are security systems that are designed to detect and respond to cyber threats. They are used to protect systems from malicious attacks, data breaches and other security incidents. An IRS typically combines threat detection, incident response and post-incident analysis capabilities to provide a comprehensive security solution.

An IRS uses a variety of techniques to detect security threats, such as Intrusion Detection Systems (IDS), firewalls and endpoint protection software. These systems are designed to identify and alert security personnel to suspicious activity, such as unauthorized access attempts or data exfiltration. Once a threat has been detected, the IRS provides a set of pre-defined response actions that can be taken to contain, neutralize and remediate the threat. These actions may include isolating infected systems, terminating malicious processes and restoring systems to a secure state.

#### 1.1.2. Identification of Malicious Traffic Using Deep Learning

Deep learning consists of the use of neural networks with several intermediate layers, with the presence of feature extraction layers [14]. Each neuron in a deep learning system has more connections and can perform more complex learning, with the ability to make more efficient predictions. Deep learning is applied to unsupervised or semi-supervised learning, in such a way that it can train, with a high level of accuracy, complex and bulky models that require higher computational costs, presenting greater performance than simpler machine learning techniques.

Deep learning has several branches and derived architectures that use neural networks to perform the complex learning steps [15]. Examples of these architectures are recurrent neural networks (RNN), used in text and video processing; generative adversarial networks (GAN), which operate by generating fake and adversarial data to distinguish them from the real ones; long short-term memory (LSTM), which has feedback connections to perform learning with the concept of memory; and also the convolutional neural network (CNN) method.

The reason for the use of deep learning in this project is to improve and automate anomaly detection by NIDS. For a NIDS to learn precisely each behavior pattern either for malicious or benign traffic, it needs to implement artificial intelligence methods. Deep learning utilizes artificial neural networks with multiple layers, being capable of learning a network dataset in a more complex manner.

#### 1.1.3. 1D-CNN

A convolutional neural network is a deep learning algorithm that is used to learn by extracting features from an input through filters, assigning importance levels to differentiate training objects and features and then performing the final classification using an activation function [16]. It is a low processing classification algorithm and can work on architectures with recurrent or feedforward training. As for the One-Dimensional Convolutional Neural Network (1D-CNN), it is commonly used to train and classify sequential data, i.e., data structured in only one dimension, such as network data captured through a sniffer.

The 1D-CNN algorithm employs convolutional layers with filters (also known as kernels) to extract features from a given dataset. These features are analyzed using matrix and operational functions to identify the most significant data points and perform feature mapping. Feature mapping plays a crucial role in ensuring the robustness of the model and simplifying data management for subsequent training steps.

The depth of feature extraction increases with the number of filters used, allowing for a more comprehensive analysis of the input data and capturing finer details. By incorporating a larger number of filters, the algorithm becomes capable of detecting more intricate patterns and features within the input data.

An important step in the 1D-CNN procedure is to perform the pooling of the data [17]. From the operational functions generated with the feature mapping, there is a simplification and reduction of the map area to perform the summarization of information. Specifically, the pooling step serves to select the main information of the filtering performed by the previous convolutional layer [17].

Finally, 1D-CNN also uses fully connected layers via neural networks to carry out the information processing by neurons in all intermediate layers. In the end, an activation function such as Softmax or Sigmoid is used to generate a classification vector and assign a category to the input that was trained from the data filtered by the convolutional layers [16].

#### 1.1.4. Federated Learning

Starting from machine learning, a derived technique for networks and distributed systems emerged, called federated learning (FL) [18]. FL also uses machine learning techniques to perform the training of a model or algorithm, but in a distributed manner among the devices of the network, so that each one uses data that are stored locally, which can be seen in a synchronous and asynchronous structure in [19]. In other words, each network device (end devices, gateways or even servers), through its own samples, performs individual training of the model without sharing information between them [18].

In this way, the learning model is trained individually by all components in an isolated manner, starting from distinct data, with each one contributing to the final version, generating the so-called global model. Thus, this technique provides training rounds via machine learning with more privacy and data security due to the fact that there is no sharing of information, besides providing access to heterogeneous data [18].

Federated learning, which can use techniques such as neural networks and deep learning to perform data classification, is applied to several nodes that will perform the training and generate parameters or weights to be used in the training process of the global model [20]. Specifically, all nodes will perform their individual training and send the results to a central server, in order to be joined and aggregated to update the main model [20].

#### 1.1.5. Fog Computing

Fog computing is a decentralized computing paradigm that extends the reach of cloud computing to the network edge, closer to the end-users and devices that generate or consume data. It enables the distribution of data, computational and storage resources in close proximity to the data source, providing a complementary alternative to traditional cloud computing architectures. Fog computing aims to address the challenges of latency, bandwidth, privacy and security that arise from the centralization of cloud computing infrastructures. Its application and effectiveness in terms of reducing energy consumption and consuming fewer data are observed in [21].

Edges devices, the nodes of an edge computing architecture, can process data remotely and can be used to help identify security flaws, analyzing traffic patterns closer to the source and eliminating the need to send all traffic to be analyzed in the cloud, thereby reducing the latency in identifying attacks [22].

In this research, the edge nodes act to mitigate denial-of-service attacks, through rules sent by the fog orchestrator. The device actively monitors traffic behavior, executed processes or other characteristics informed by the orchestrator and, finding patterns classified as malicious, it applies rules, which are simply sets of actions in the operating system, to stop participating in the botnet or sending malicious traffic.

## 2. Related Works

Botnets and DDoS attacks have become a matter of great concern, especially considering the continually increasing number of devices connected to the internet and the sensitive information about the real world carried by these devices.

Within this scenario, this work considers papers that investigate IDS and IPS for IoT devices, either as a victim or as an agent of attacks. We discuss the different approaches that have been taken referring to this issue throughout this section.

### 2.1. IoT Intrusion Detection Systems

IoT devices are generally resource-constrained in terms of their processing, storage and power supply capabilities. Thus, they are usually small devices that can operate in the most diverse places and conditions.

The majority of IDS implementations in IoT are based on adapting existing detection tools to devices usually used in the IoT context and managing the necessary resources. Our proposal in the present work, however, is based on the creation of a Host-Based Intrusion Detection System specifically for the IoT context and with a set of rules focused on common IoT vulnerabilities.

The heterogeneity of the technologies and configurations of IoT network components is another issue that makes the countermeasure more difficult to apply and to be useful in IoT networks. Some papers propose specialized solutions that are limited to their specific technology or protocol. The authors in [23] propose an IoT device to monitor and analyze the network DNS traffic, verifying whether any anomalous pattern is detected in its queries and responses. The work [24] proposes an IDS for IoT devices that communicate by 6LoWPAN in IPv6.

Other researchers focus on how to detect intrusions that may have occurred in any host. The authors in [23] implement their own anomalous behavior detection mechanism by analyzing the DNS queries that are transmitted through the network. The researchers in [25,26] employ complex event processing (CEP) to analyze the IoT network data stream and its monitoring of any security events. According to [27], agents collect network and device behavior data and then apply some artificial intelligence techniques, such as artificial neural networks in the former and a naïve Bayes (NB) classifier algorithm in the latter. In [28], the authors suggest the Abnormal Behavior Analysis Methodology, in which the network nodes’ operation is monitored and compared to regular operation. Then, they trigger actions when abnormal behavior is detected.

We recognize the importance of these intrusion detection proposals; however, our work does not focus on the intrusion detection mechanism. It focuses on providing the host with an analysis engine, as well as on taking the necessary measures for prevention and repair.

### 2.2. Host-Based IDS for IoT Devices

Host-Based Intrusion Detection Systems (HIDS) are Intrusion Detection and System (IDS) software programs designed to run only within a target host, where the HIDS performs the tasks of monitoring and analyzing files, memory, input/output and processes [29].

Studies of HIDS address different detection and mitigation techniques, machine learning algorithms, system architectures, signature rule utilization and anomaly identification, among other important aspects for the implementation and use of these tools.

The work presented in [30] designed a Hybrid Intrusion Detection System (HIDS), using signature- and anomaly-based methods in its structure. The work shows that the hybrid solution with signature- and anomaly-based detection presents greater accuracy than when using only one of the methods. Using the Bot-Iot dataset, one of the work’s main goals was to detect zero-day vulnerabilities with low false positives through a high detection rate using a hybrid approach with signature- and anomaly-based detection.

Considering that the anomaly method is activated after the signature method identifies that it is a new attack, we show a different method, because the IDPS is applied in the IoT device after traffic anomaly detection using distributed artificial intelligence. Before attack mitigation, our proposed solution first detects whether a bot is infected or not.

### 2.3. Deep Learning for Network-Based IDS

Network Intrusion Detection Systems (NIDS) are security tools designed to detect and respond to network-based attacks. These systems continuously monitor network traffic and analyze it for indications of malicious activity, such as attempts to exploit vulnerabilities or unauthorized access. NIDS can be deployed at various points within a network, including the perimeter and internal segments, to provide layered security and enhanced visibility regarding network activity.

In the context of IoT, anomaly-based intrusion detection methods using artificial intelligence, particularly machine learning, are gaining popularity due to their effectiveness in identifying abnormal behavior in network traffic. Deep learning, which involves neural network architectures, has emerged as a prominent approach in Intrusion Detection Systems (IDS), as it leverages the power of fully connected layers containing neurons that can learn patterns from network data.

The primary objective of deep learning is to recognize, differentiate and classify unknown data by utilizing complex learning processes with hierarchical layers that extract key features from network traffic. Through neural networks, deep learning algorithms can make intricate and accurate predictions using intricate models by extracting deep features from the data.

Recent works, such as [31,32], use an algorithm called the One-Dimensional Convolutional Neural Network (1D-CNN), which includes convolutional layers, i.e, layers capable of extracting the most vital features from sequential input data (one dimensional) and selecting them to perform accurate classification afterwards. Its importance is related to the necessity of improving the detection performance of AIDS, especially considering the emergence of new zero-day attacks on IoT networks.

The work [31] proposes a Network Intrusion Detection System (NIDS) with deep learning methods. The deep learning methods used are 1D-CNN and long short-term memory (LSTM), to classify multi-categorical captured data in a cyber-attack scenario. The authors suggest an architecture containing a FreeBSD firewall with signature-based IDS software to capture anomalous data in real time. Then, a learning dataset is built through generated logs and trained through deep learning for active anomaly detection. Their approach was tested in a six-category multi-class dataset, and they showed good performance when detecting anomalous data between benign traffic, especially when comparing its results with regular machine learning and neural network algorithms.

Another similar work is presented in [32], which applies a 1D-CNN-based IDS with an unbalanced and balanced UNSW NB15 dataset using a different number of 1D-CNN layers in each iteration. The authors present performance comparisons with regular machine learning methods, such as random forest (RF) and support vector machine (SVM), showing that the 1D-CNN solution gives higher accuracy, recall and F1 scores when it trains on balanced datasets, in comparison with the RF and SVM algorithms.

The work [33] involves the use of an Intrusion Detection and Prevention System in a cloud computing infrastructure. Its application, similar to this project, involves the use of a NIDS and a HIDS, forming a hybrid architecture. The architecture uses the K-nearest neighbor and neural network (KNN-NN) algorithms in the cloud, to train and classify network data from the NSL-KDD dataset. Thus, the objective of this work is to structure a Cloud-IDS with hybrid algorithms to classify network anomalies in a large data flow in the cloud.

### 2.4. Federated Learning for Network-Based IDS

Recent works on NIDS for IoT, such as [34,35], study a distributed and decentralized machine learning implementation between multiple clients with different architectures (hybrid structure), called federated learning (FL). This method involves the usage of a central server responsible for coordinating the training process through multiple rounds. In each round, the server sends a global model (a trainable dataset) to all different devices, which are present in the architecture as federated learning clients.

The main goal of the devices is to apply a learning algorithm, which could be performed through deep learning, able to train the dataset locally and individually. The clients send the results back to the server responsible for aggregating them and building a final model. Therefore, federated learning is an important method used to improve privacy and security during deep learning, using training contributions from different types of devices, especially IoT endpoints.

The work [34] proposes an IDS with federated learning and edge computing, called FedACNN, which uses a CNN deep learning model in each IoT device responsible for training a public dataset called NSL-KDD. The authors utilize an aggregation algorithm called federated averaging (FedAvg) and try to reduce the communication delay through an attention mechanism that allows a lower detection delay between each federated learning round. The results show high training and testing accuracy, including a reduction of 50 percent in the communication rounds.

The work [35], also called DIoT, discusses the implementation of an IDS with federated learning based on device-type-specific detection models. It identifies anomalies through the ways in which devices communicate concerning their structure and behavior and then performs federated training based on what is detected. The authors used a botnet to inject malicious traffic into the network and applied a federated training method to identify each attack through 30 IoT devices based on their models.

### 2.5. Fog Computing

Fog computing is a technology that enables data processing on edge devices, such as routers, switches and hubs, instead of relying on centralized resources in remote data centers. This approach can help to reduce latency and improve data security and privacy, as well as reduce the network bandwidth consumption. With the increasing use of IoT devices and the demand for real-time security measures, works are using fog computing in IDS for anomaly-based attack detection and mitigation.

The fog-computing-related work [22] implements an anomaly mitigation system for IoT to prevent DDoS activity using fog computing in order to address botnet threats. This work uses three statistical algorithms, namely exponentially weighted moving average (EWMA), K-nearest neighbors (KNN) and the cumulative sum algorithm (CUSUM), to detect malicious botnet activity in IoT networks. Using the Bot-IoT dataset, it obtained 99% accuracy during anomaly detection and was able to differentiate non-IoT from IoT devices.

### 2.6. Technologies Summary

In Table 1, we describe all technologies described in this section to highlight their advantages, disadvantages and application scenarios. This work presents contributions regarding all technologies mentioned in terms of security, privacy and anomaly detection measures, as described in Section 1.

## 3. Proposed Solution

**Figure 1:**
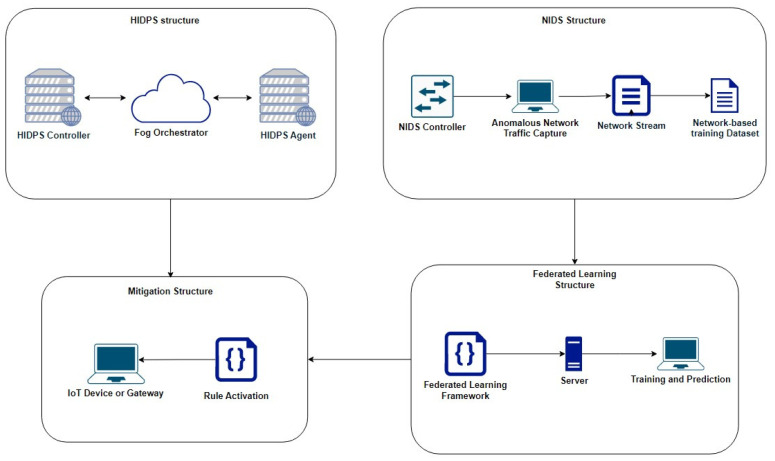
Logical architecture of the proposed hybrid solution.

This paper presents a hybrid detection and prevention architecture, as shown in Figure 1.

This architecture is divided into the HIDPS structure, the NIDS structure, the federated learning structure and the mitigation structure. It contains a signature-based distributed Host Intrusion Detection and Prevention System (HIDPS) and an anomaly-based distributed Network Intrusion Detection System (NIDS) that, when integrated, form an Intrusion Response System through anomaly detection using a distributed learning method called federated learning.

First of all, the HIDPS solution used in this work was developed with the goal of mitigating signature-based denial-of-service attacks and can be applied to IoT devices or gateways [6]. Anomaly signatures are defined by an administrator in a fog computing orchestration portal, queried periodically by hosts [9]. The HIDPS copies the rules to its local base, where they contain the behavior that must be observed and the action required to carry out the mitigation.

The HIDPS will be able to receive signatures from the fog orchestrator, indicating the behaviors and traffic that must be mitigated, and the detection of anomalies will be performed by analyzing the network traffic.

In the NIDS structure, the captured traffic will be exported to a stream, which can be from different sources, such as port mirroring. The received flows will be processed by a decentralized network of nodes, responsible for classifying and identifying hosts that are infected by a botnet and promoting denial-of-service attacks.

The federated learning structure will distribute the traffic-based dataset using an adversarial model as a pre-configured botnet to hybrid clients coordinated by multiple servers. These clients will use the 1D-CNN deep learning method to individually classify unknown events based on their nature.

We position the edges and the federated learning server on the local network, bringing the benefit of reducing data transmission to external networks. Another benefit of this model is that if the signatures defined in the HIDPS orchestrator fail and the IoT devices initiate a denial-of-service attack, the same will be detected by the anomaly caused.

We developed a module that interconnects the federated learning server with the IoT devices that use the HIDPS software, making it capable of triggering rules to interrupt the data flow identified as anomalous. For this work, we developed an interface to interconnect only the server with the HIDPS software.

Finally, using the mitigation structure in order to connect both IDS structures and to build the finished version of the architecture, the federated learning component of the NIDS will be responsible for detecting and classifying captured events to build a final model in the central server, capable of generating new operational rules to be used in the HIDPS, especially in prevention mode. Therefore, the NIDS segment will work in parallel with the HIDPS, updating its database with new rules generated through malicious activity pattern recognition in distributed deep learning.

**Figure 2:**
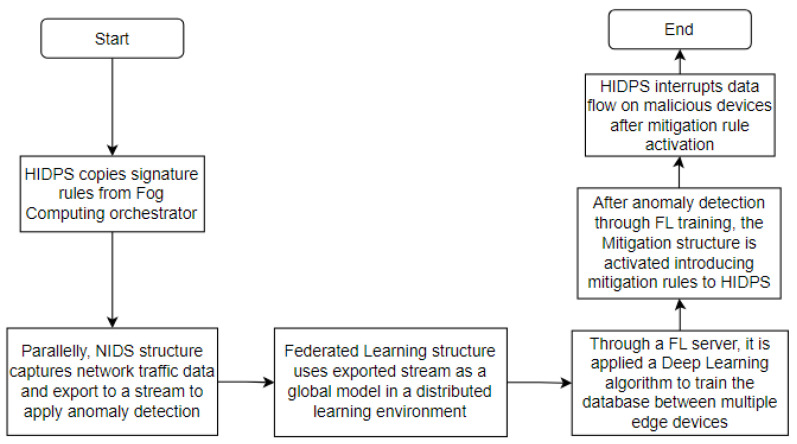
Logical flowchart summarizing each structure’s activity in the proposed hybrid solution.

In order to summarize all activities performed by each structure in the proposed solution, as explained before, Figure 2 shows a flowchart emphasizing the functions and interconnections between the architecture’s segments presented in Figure 1.

In this section, the architecture is separated into the following entities: the HIDPS structure, the NIDS structure, the federated learning structure and the mitigation structure.

### 3.1. HIDPS Structure

**Figure 3:**
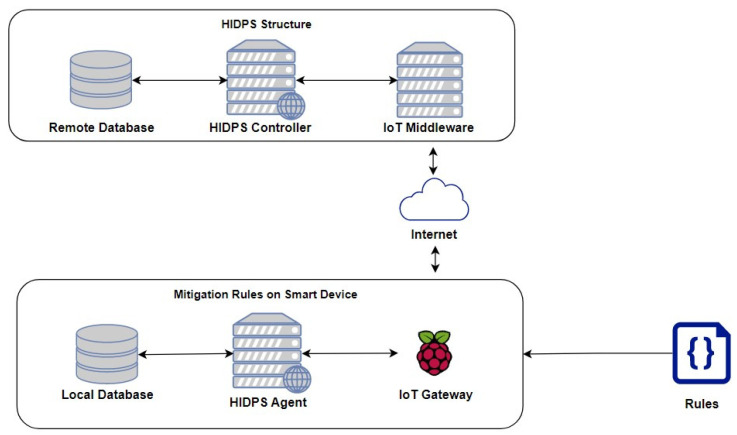
Logical architecture of the IoT HIDPS structure.

The proposed distributed HIDPS for IoT has several components. As shown in Figure 3, the proposed HIDPS comprises distinct entities that aim to detect attacks and vulnerabilities in smart devices connected to a given IoT instance.

In this logical architecture, several components are used: a HIDPS agent running on a smart device, a local database and a HIDPS controller with a remote database communicating with the HIDPS agent through an IoT middleware over the internet, being executed by an API.

The HIDPS agent, which will run on an IoT smart device, has a local database containing local rules that will be used to trigger and send events to the HIDPS controller from the internet. The HIDPS controller manages the HIDPS agents by updating, inserting and maintaining rules in the agent’s local database from its remote database. The IoT middleware serves as a method of communication between the HIDPS controller and a smart device acting as the HIDPS agent.

Thus, Figure 3 represents the logical communication of the HIDPS structure used in this project on a real IoT network.

The HIDPS agent, fog orchestrator, HIDPS controller and rules are described hereafter.

#### 3.1.1. HIDPS Agent

The HIDPS agent process is executed as a service on the IoT gateway and devices and evaluates the test defined by the rules on its local database. The evaluation output is then compared against the expected output. If the output coincides, the action specified in the rule is executed, and an event report is generated and sent to the HIDPS controller.

The HIDPS has a threat scan function that refers to the rules registered on the local database and executes test cases for each rule. The test case’s objective is to verify that the output is the same as that expected by the signature rule registered; if the output coincides, the activities related to the rule are executed, and an event report is generated and sent to the HIDPS controller using the communication API.

The agent can enable and disable the self-analysis execution. When enabled, the threat scan is performed automatically and periodically.

To fetch rules from the HIDPS controller, the agent has the update rules function, which requests the controller web server via the communication API. When the HIDPS agent receives the set of rules, the HIDPS application compares the fetched set of rules against the local database, and if any rule has been added, updated or removed from the previous set, the agent will update the local database.

The rule updating process can be executed through the Command Line Interface (CLI), but the HIDPS application automates this process by running it automatically and periodically. The time between updated requests can be updated by the administrator through the set the time between rule updates function. If the time interval is not configured, the time associated with the process will be 15 min.

The system administrator also has access to the function that allows him to inspect the agent configuration. The show rules function lists all the existing rules on the HIDPS’ rule table on the local database. The function show settings returns the HIDPS application settings, such as the updating time interval and the configurations of the communication API.

#### 3.1.2. Fog Orchestrator

In order to send signature rules and event reports between the cloud and the end devices with the HIDPS agent, a fog orchestrator is used to insert and update rules into the agent’s local database. Figure 1 shows that a fog orchestrator, to connect the cloud IoT and smart devices, composes the HIDPS structure to inform agents about which behavior and traffic need to be mitigated through a set of signatures [9].

The cloud IoT, shown in Figure 1, composed by the HIDPS controller, has a remote database containing the signatures that need to be inserted into each edge device’s local database. The fog orchestrator, managed by an administrator through a portal, defines signature rules after each query made by the end devices. Each smart device periodically sends queries in order to apply new rules that will be used in attack mitigation.

Therefore, a fog orchestrator is applied between the HIDPS agent and HIDPS controller to control and manage the signature queries that update each agent’s local database. This method is introduced to reduce latency and bandwidth costs in order to reduce significantly the rule time consumed by each end device.

#### 3.1.3. HIDPS Controller

The network has a HIDPS controller that manages all HIDPS agents configured in each IoT device. The controller can log and update detection rules, define rules’ premises, analyze event reports and provide data visualization to the administrator. The HIDPS controller has a direct channel with the IoT gateway and can define customized assumptions and report the findings. The rule registration process can be performed by an application running on the server or via the web API. Some of the functions are described as follows.

The HIDPS controller has a function to register new rules on its local database. The rules on the database are mirrored to the web server page and then updated by each HIDPS agent.

The definition of assertion function defines the expected output from the test case that is executed by the HIDPS agents to verify the existence of threats and vulnerabilities.

The HIDPS controller has the function event report analysis, which is continually analyzing the event reports that arrive at the HIDPS controller coming from the HIDPS agents associated with it.

The event report analysis function analyzes the event reports received by the HIDPS controller coming from the HIDPS agents’ rule execution.

Based on the event report, the threat treatment performs some action to counter the reported vulnerabilities.

#### 3.1.4. Rules

A rule is an information block that assists the HIDPS agent in taking some previously defined actions, an abstraction that has the following six main fields.

- Name: Rule name;
- ID: Uniquely identifies a rule;
- Date: Date of creation;
- Assumption: Value taken as accurate for a given condition or characteristic of the system;
- Test case: Evaluation code that finds the characteristic or condition to be tested and returns it for verification;
- Action: If the value found in the test case is different from the value found in the assumption, the action defined in this field must be taken.

The registered rules must prevent anomalies commonly found in IoT systems. For the sake of simplifying the application, these rules are classified into different contexts: network, resources and known vulnerabilities.

In the context network, the HIDPS agent’s connection with the IoT gateway is obtained through a traditional network infrastructure that potentially presents several well-known vulnerabilities and attacks. These vulnerabilities can serve as entry points for malicious agents to access and control the device that hosts the HIDPS agent. Thus, the HIDPS rules were developed, intending to detect vulnerabilities and attacks related to the network context, including, for instance, those shown in Table 2.

In the context of resources, it is essential to be concerned about these, as smart devices have resource constraints. Thus, overloading of these resources can represent a target of attacks that aim to reduce the resource allocation for legitimate applications. To detect this type of attack, rules based on this context have been created, including the ones presented in Table 2.

An awareness of the context of known IoT vulnerabilities made it possible to create new rules based on these vulnerabilities, specific to IoT environments and regarding smart device configuration best practices. Rules implemented in this context are described in Table 2.

To report to the HIDPS agent when a detection occurs, an event report is established in the HIDPS agent. The event report is an information schema created by the HIDPS agent and shared with the HIDPS controller. This report is generated and sent when a vulnerability is detected by the rules present in the HIDPS agent.

The HIDPS agent uses an algorithm that defines the behavior of the application on the smart device that wishes to send data to the HIDPS controller cloud application that will handle the received data.

**Figure 4:**
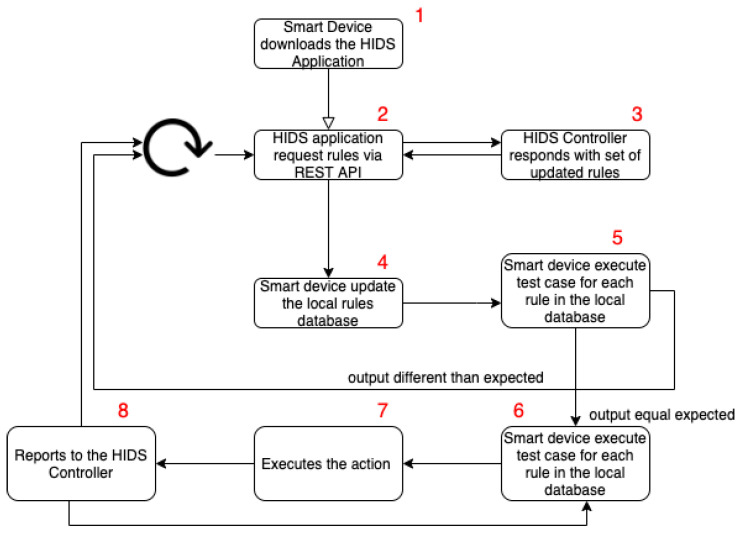
Application life cycle from the perspective of the HIDPS agent on a smart device.

The HIDPS agent needs to perform the algorithm, step-by-step, to detect, track and treat vulnerabilities and attacks. The algorithm steps shown in Figure 4 define its life cycle. We present the actions performed by each HIDPS agent, at the moment that it needs to send data to the HIDPS controller cloud application, which will handle the received data as follows:

1. Register the HIDPS agent on the HIDPS controller;
2. Download the HIDPS source code;
3. Request updated rules to the HIDPS controller;
4. Validate the HIDPS agent request and respond with the updated rules;
5. Update the local rule database;
6. Runs preventive tests for each local database rule;
7. Perform the actions provided in the rules;
8. Send event report;
9. Handle received event reports.

The first step is to register the HIDPS agent on the HIDPS controller. If the smart device is new to the IoT network, and it is due to enter it, it needs to register itself in the IoT middleware to be able to send data to the middleware and also interact with other devices. Thus, the device must perform its self-registration process in the UIoT, as described in Section 2. After completing this process, the IoT middleware considers this new device ready to interact with the network. Then, as soon as this process is completed, the smart device will be registered in the HIDPS controller as an associated smart device.

At the end of the self-registration process, the IoT middleware sends the HIDPS agent to be installed by the smart device. Then, the device should download the HIDPS agent and install and implement it. From this point, the smart device runs an instance of the HIDPS agent and can be accepted as a valid device in the IoT network instance. It is important to mention that the IoT middleware blocks all IoT devices without a running instance of the HIDPS agent.

Since the smart device is registered and set to work, it can begin to send its sensor data to the middleware, as with any other regular device in the network. During its normal lifetime, it can perform rule updating and evaluate the device status. Both events can be triggered by a scheduled job or by the CLI.

Occasionally, the HIDPS agent requests updated rules from the HIDPS controller. It makes this request using its local communication API and waits for the controller’s answer.

The HIDPS controller validates the requesting HIDPS agent based on its identification, previously registered, during the self-registration process. Thus, the instance is validated, and then the HIDPS controller responds with the set of updated rules.

Once the updated rules have been received in the HIDPS agent, it updates the local rule database.

As the local rule database is updated, the HIDPS agent runs preventive tests for each local database rule. Thus, each rule is tested to evaluate whether the resulting output is different from the one expected for each specific rule.

For each rule that fails on the test runs, the HIDPS agent will perform the actions provided in the rule.

For each testing mismatch result, the HIDPS agent sends the event report to the associated HIDPS controller. Through the event report, the administrator can evaluate the status and execute actions on the HIDPS agents.

The HIDPS controller handles the event report received. First, it stores this event in the database. Then, it performs actions to counter the vulnerabilities reported by the HIDPS agent. An example action would be to create IPTables rules to interrupt the connection between a device and any other action that the administrator evaluates as correct, to stop any irregular behavior.

### 3.2. NIDS Structure

**Figure 5:**
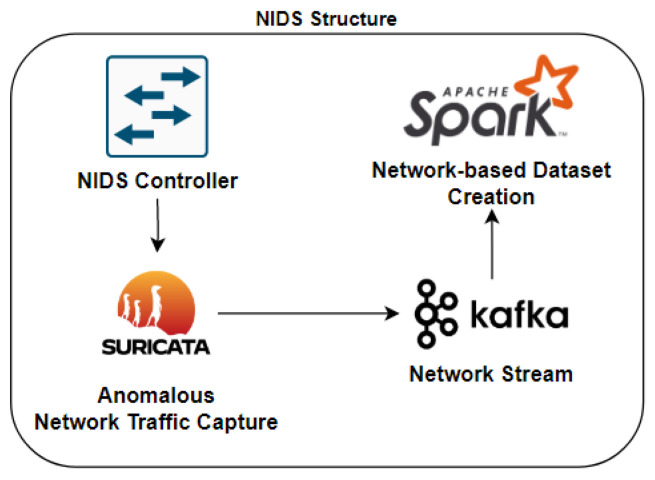
NIDS proposed structure.

The proposed distributed NIDS for IoT has components related to previous strategies. As shown in Figure 5, the NIDS comprises entities that detect attacks and vulnerabilities in smart devices connected to an IoT instance. The process begins with the injection of malicious data using a botnet, monitoring the network with an IDS sniffer and building a traffic dataset with data processing and transformation software. The dataset is then used in a federated learning structure with deep learning methods to classify previously captured network events. The NIDS controller, anomalous network traffic capture, network stream and network-based training dataset are described hereafter.

In general terms, the solution presents pure network monitoring through signature-based methods and the creation of a machine learning dataset to classify packets as anomalous or benign data.

#### 3.2.1. NIDS Controller

The NIDS controller is responsible for capturing all network data generated in an IoT network created by smart devices and a pre-configured botnet. The controller consists of an IDS sniffer and real-time data processing software to structure a machine learning model to be used in a federated learning system.

The capturing method uses a switch with port mirroring to send copies of network packets from source ports to a destination interface, enabling the capture of all traffic running on the switch device. The NIDS controller captures the traffic between all smart devices and the botnet, building a machine learning model for training and detection purposes.

The capturing process is performed by the Suricata IDS/IPS software using signature rules with flow metrics and patterns to capture running network data. Consequently, a distributed publish–subscribe messaging system called Apache Kafka processes the information, and a data transformation application called Apache Spark structures the network dataset.

#### 3.2.2. Anomalous Network Traffic Capture

To capture malicious network data in the network, IDS/IPS software is used in the NIDS controller, which is called Suricata. Suricata is an open-source software program that analyzes and detects threats in a local network using a set of pre-established rules. It supports the IDS and IPS in traffic monitoring and performs deep packet inspection using multi-threading to handle a high volume of data. In addition, Suricata integrates with multiple applications, performs protocol transactions and can extract complex files.

In this work, the network-monitoring-based software is utilized to actively detect data through all ports in the switch responsible for applying port mirroring on the NIDS controller. This method implements a capturing log called Eve JavaScript Object Notation (JSON), which structures packets—containing network, signature and flow information—based on multiple fields. Each field is customizable and refers directly to network data characteristics that are important to analyze and use as training criteria during the federated learning process.

**Figure 6:**
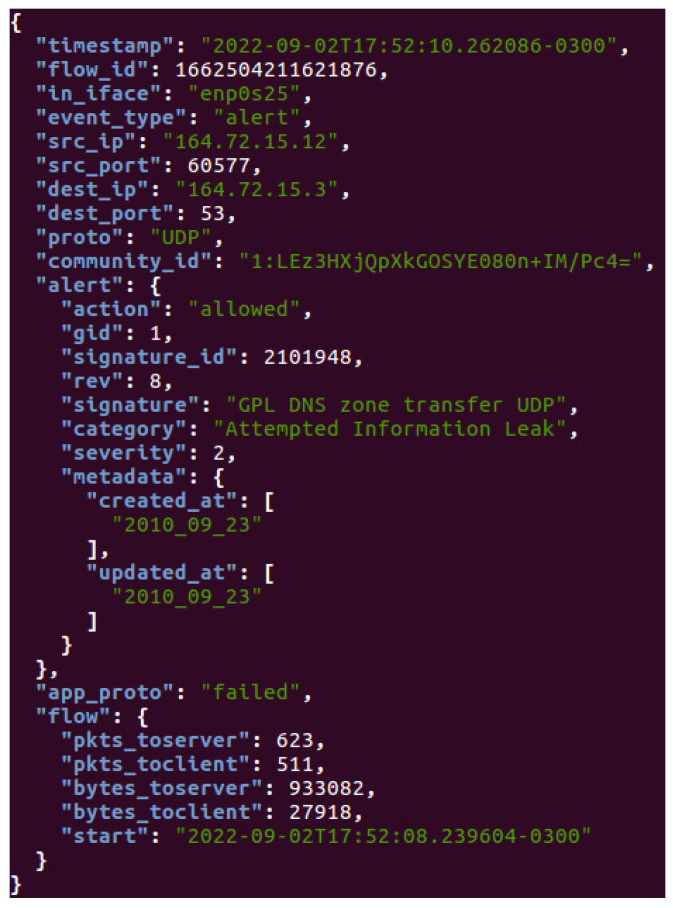
Captured Suricata log in JSON format.

Therefore, as shown in Figure 6, the Suricata software is responsible for collecting all network activity to generate a JSON log containing essential network, signature and flow information about each captured packet. Consequently, this information is extracted by a data transformation application to create a machine learning dataset.

#### 3.2.3. Network Stream

To use the network stream to generate a model dataset, Apache Kafka will be used in this proposed model. Apache Kafka is an application that works as a messaging system for logs or events, containing a data flow service in such a way that these are processed at the destination, i.e, in real time. Kafka works with logs as in its storage and works with a large volume of data, so it is scalable, distributed and works at a high speed. Its operation is based on writing (publishing) data and reading (subscribing) data to an application.

Its architecture consists of a Kafka cluster having servers called brokers, consisting of topics (or partitions) that will either receive or send data during data processing. The producers will publish and send the data to the topics in the cluster, with the writing of messages from some application external to Kafka, and the consumers are applications that will receive the messages directly from Kafka topics.

**Figure 7:**
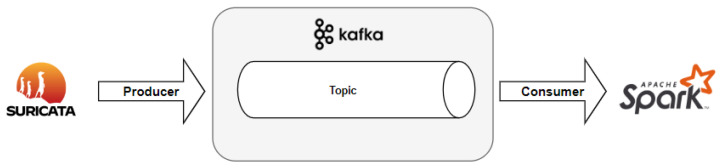
Consumer and producer diagram of Apache Kafka.

Therefore, Apache Kafka is used as a bridge between Suricata and the data-transformation-based software called Apache Spark. Each log generated during network traffic monitoring by Suricata will be written in a Kafka topic—acting as a producer—merely to be consumed by Apache Spark later on (acting as a consumer). As shown in Figure 7, Apache Kafka acts as a messaging system between network data capturing and machine learning dataset creation.

#### 3.2.4. Network-Based Training Dataset

To use the Network Stream to perform the training, detection and prediction of anomalous data, a training dataset will be structured to act as a global model in the federated learning procedure. This dataset will use all flow and signature parameters implemented by Suricata during network traffic capturing. Then, using a software program called Apache Spark, an ML dataset will be generated with all Suricata’s parameters as training features.

The Apache Spark software is a tool for the processing, transformation and analysis of data in real time and in a distributed form, using SQL, data science and graph processing and applying machine learning to data streams.

To be able to perform data transformation, Apache Spark has three types of programming languages that can be used: Java, Python and especially Scala. The latter will be used in this project, bringing the possibility of performing filtering, mapping and reduction functions, among others. From these tools, it is then possible to generate datasets (following the structure of columns and values in a table) from various file formats, such as Comma-Separated Values (CSV), JSON and Parquet, among others.

The goal of Apache Spark in this project is to consume the network capture data coming from Kafka to use the programming component—made in Scala—and transform, in real time, the information in JSON format into a dataset. Each field of the log will be transformed into a column containing the values of events that were previously recorded by Suricata. In other words, Spark has the function of generating a dataset from the logs created by Suricata. This step occurs mainly through the integration between Apache Spark and the Kafka API from the structured streaming component by reading the stream contained in the Kafka cluster topic in real time.

Using the Scala programming code with direct integration between Apache Spark and Apache Kafka, the cluster will receive each log in JSON format and transform each field into a column with its respective values. The generated columns will be written in a CSV file, which can be easily read by a machine learning module and then used by the federated learning structure to continue the detection process.

Through the Scala code implemented by Apache Spark, a dataset was created in CSV through the SQL language to structure all flow and signature data into features. The dataset generated in the NIDS structure has 15 active features and they are described in Table 3.

### 3.3. Federated Learning Structure

**Figure 8:**
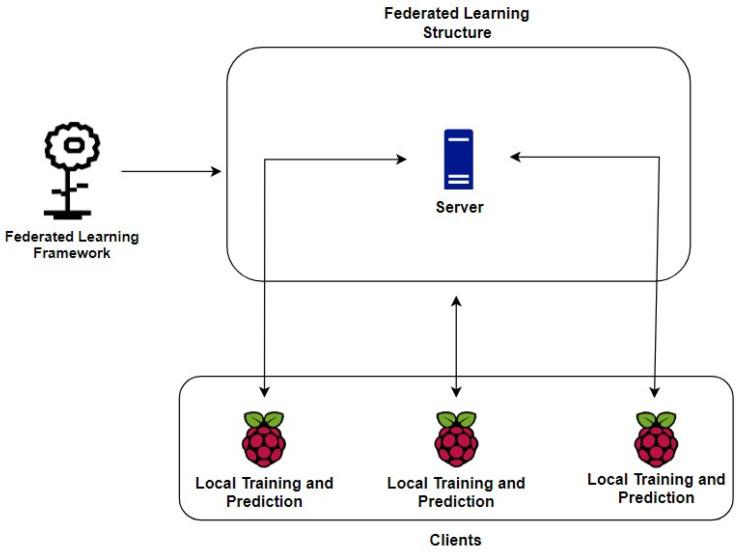
Federated learning proposed structure.

After the successful capture of the traffic generated in the local network, having data regarding the actions of the Mirai botnet, the training model previously trained is used to implement its training by machine learning and to perform the classification of the newly captured packets to identify anomalies. The training technique will be implemented through federated learning in an environment with one server or multiple servers and multiple clients, to perform the entire learning process in a distributed way and with a higher level of privacy. The federated learning structure is shown in Figure 8.

**Figure 9:**
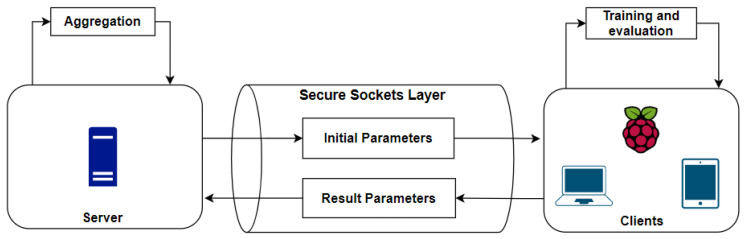
Federated learning connection between server and clients diagram.

As shown in Figure 9, federated learning performs the training locally on each predefined user, i.e., they apply individual training, having performance differences among the other clients, in such a way that the final model will have contributions from all participants. The entire training process will be coordinated by a server that will have all the learning strategies, i.e., all the methods to be used, such as how to aggregate the results, how to pass parameters to clients, the number of clients that will participate, the number of rounds and the number of epochs for each client, among other characteristics to be defined later.

The clients will possess the model locally, and by dividing the training sets and learning via the One-Dimensional Convolutional Neural Network (1D-CNN) deep learning algorithm, they will perform the entire procedure of training and evaluating the dataset. To improve the communication security between the server and the clients, a Secure Sockets Layer (SSL) protocol is implemented between them in such a way that each participant needs to be authenticated through a digital certificate, thus increasing the privacy during all training processes. The federated learning framework, server and training and prediction are described hereafter.

#### 3.3.1. Federated Learning Framework

In order to define the federated learning structure on the server and clients, the project utilizes the Flower framework. Flower is an open-source and easy-to-implement tool that integrates Python with various federated learning languages, libraries and learning methods, such as TensorFlow, Skicit-learn, PyTorch and Numpy.

The framework [36] was chosen because it provides all the necessary resources to achieve the desired training objectives and offers high modifiability and customization options, enabling the implementation of different strategies. It is a versatile framework that includes a wide variety of training and model evaluation implementations. Additionally, it can be applied in cloud computing infrastructures, mobile networks and IoT networks, making it possible to perform training on Android devices, the Raspberry Pi and the NVIDIA Jetson.

The Flower framework enables the construction of a federated learning structure with a server and multiple clients, while offering various strategies and implementations to enhance the training performance, client coordination, security, privacy measures and more.

The main library used in the learning code will be TensorFlow with Keras, including libraries such as Pandas and Skicit-Learn to perform dataset manipulations, with the presence of the 1D-CNN algorithm to conduct the training from the CSV dataset generated by Apache Spark. To validate and evaluate the performance of each client in terms of accuracy and loss, the server contains all possible strategies for training, evaluation, security, privacy and aggregation measures, which are described hereafter.

- gRPC Server: A remote procedure call framework that provides communication between the server and clients.
- Secure Sockets Layer: An encryption-based security protocol.
- Server-side parameter initialization: The server is responsible for initializing the training parameters.
- Server-side aggregation by fault-tolerant federated averaging: The server is responsible for obtaining all result parameters from clients after the training process, to aggregate them and generate a final global model. The fault-tolerant FedAvg algorithm aggregates all result parameters from clients by taking the weighted average and can tolerate faulty client conditions such as client disconnections or failures.
- Sending/receiving arbitrary values to/from clients: The server is responsible for sending configuration parameters to clients, such as the number of rounds, number of training epochs and batch size.
- Federated evaluation: A certain number of clients are selected to evaluate the final model after the aggregation process by the server.
- Saving progress: This is a server-side strategy responsible for continuous model updates in such a way that preserves the training model. It can be used to implement new rule generation structures during the hybrid IDS approach.
- Input data prediction and classification: Each client uses the trained model to classify new input data sent by the server to determine the attacks that were captured by the NIDS.
- Federated HIDPS rule activation: All clients classify a new input model after the training procedure and then utilize evaluation metrics in order to activate the HIDPS rules.

#### 3.3.2. Server

The server plays a vital role in ensuring the efficient and secure execution of the federated learning process. It is responsible for defining training strategies for clients, establishing communication with them through a gRPC server, creating an SSL security layer by generating certificates, sending training parameters along with an initial evaluation and aggregating the results of subsequent training. The server’s code is written in Python and utilizes the TensorFlow and Keras libraries from the Flower framework.

One of the key responsibilities of the server is to manage client selection for each step of the federated learning process, including initialization, fitting and evaluation. By utilizing the previously implemented strategies, the server can determine the number of clients required to initiate the federated learning process and assign specific tasks to a fraction of clients, enabling a highly customizable training process in terms of management.

The central server is responsible for initiating all processes and coordinating the activities of each client based on strategy specifications, such as configuration parameters and round administration, among others. The communication steps required to initialize the server and commence the training process with the clients are based on the guidelines provided in the Flower framework documentation (referenced as [36]).

#### 3.3.3. Training and Prediction

The federated learning edges, or clients, will be initialized through their Python codes in such a way that they will be running on each Raspberry Pi, used as an experiment in this project, being different devices from those infected by the adversary model. Uninfected Raspberry Pi devices were used as the edges of the federated network, as a means to validate the use of devices with a lower processing capacity, thus allowing this model to be executed in environments with more limited computational resources or on servers with greater processing power.

Clients will receive the dataset in CSV format and apply their training individually, without interfering with one another. To connect to the gRPC server securely, they will use the certificate that was generated by the server when it was executed the first time. When they successfully connect, they will each receive the initialization and configuration parameters from the server to start their individual training through a deep learning algorithm.

Each client can either perform the fitting or evaluation task, depending on the server-side pre-configurations. After each task, the client sends back to the server the updated weight parameters to be aggregated and training and evaluation metrics such as accuracy and loss. In addition, each client generates test results during the prediction and classification steps for non-classified data through metrics such as precision, recall and the F1 score, which describes the classification performance of all attacks made by the adversary model.

To train the generated dataset in a distributed infrastructure, it is applied a deep learning algorithm based on 1D-CNN in each federated client. First of all, this algorithm extracts the best set of pattern information about the dataset in order to perform complex training in the fully connected hidden layers, which are capable of exchanging characteristics through the result parameters between neurons. At the end of the algorithm, an activation layer differentiates each event into multiple categories from its behavior patterns.

**Figure 10:**
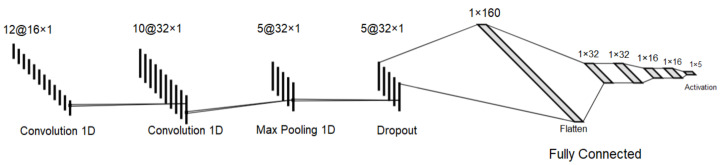
Proposed solution’s deep learning 1D-CNN model structure.

As shown in Figure 10, the algorithm’s structure has two convolutional layers responsible for extracting information, a pooling layer responsible for selecting the best row of recognized patterns and multiple connected layers responsible for removing unimportant information, continuing training and reducing overfitting, until the final classification step performed by an activation layer.

All filter sizes correspond to the dataset’s input shape after its startup, also indicating all used parameters in each layer during the training and fitting process. After its compilation by each client, the test partition is classified and evaluated through performance metrics, which are explained in the Results section. This model is used in fitting methods containing specific configuration parameters, such as the validation split, verbose mode, batch size and epochs.

### 3.4. Mitigation Structure

**Figure 11:**
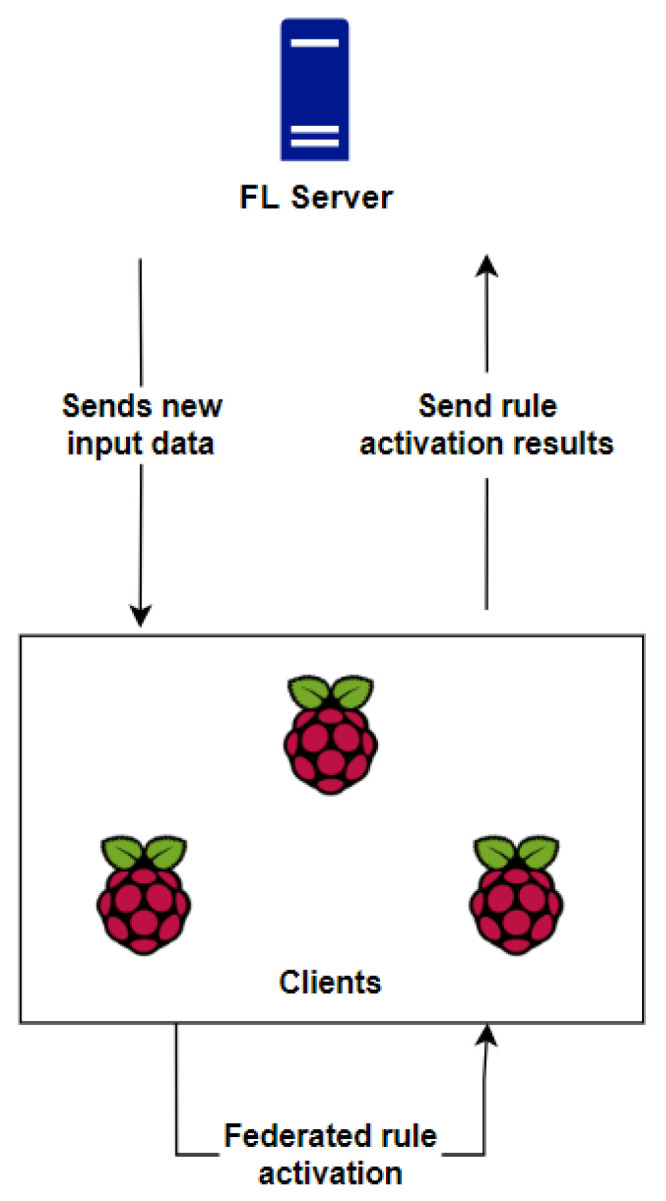
Federated mitigation rule activation diagram between server and clients.

Figure 11 demonstrates the logical diagram of the federated rule activation process, showing the relationship between the server and the clients during the classification and detection process.

The server has the role of sending a new unclassified model, obtained from the active capture of the detection model in the IDS controller, to each of the clients. The goal of sending a new capture is to actively detect network traffic after its capture. Therefore, edges will predict and classify each event based on the training of the base model, i.e., following the knowledge, in terms of behavior patterns, learned by each edge.

Then, after predicting the new events, each edge will use the classification answers to check which rules should be activated by the HIDPS in order to perform the proper preventive actions. Each edge performs this task through a shell script, so the threshold metrics used are the accuracy and loss values during training. These values are used to determine whether it is valid for the edge to determine which rule should be activated and whether its classification is reliable. Then, from what was detected after the classification, each edge individually determines which rules should be activated by the HIDPS.

At the end of the federated activation, each edge sends its results back to the server. The server tries to reach a consensus among all the proposed rules to determine which ones should be activated by the HIDPS. After the evaluation, the server decides which rules should be activated and integrates them with the HIDPS in order for it to apply preventive actions from its database.

**Figure 12:**
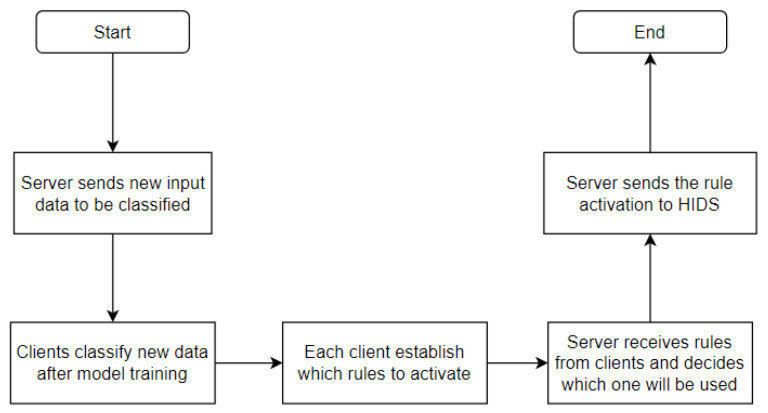
Federated mitigation rule activation flowchart between server and clients.

Figure 12 shows the step-by-step process by means of a flowchart.

## 4. Methodology

To validate the proposed model, an isolated local network was created, consisting of a botnet with devices infected by the Mirai software and its controller running on a local server. This network was set up in an isolated environment to prevent a massive infection and to ensure that the academic environment was not affected by excessive denial-of-service traffic.

The Mirai botnet was chosen due to the ease of obtaining its source code and controller configuration. By creating a local botnet, it was possible to accurately measure the response time to mitigate a denial-of-service attack.

The validation of the proposed solution involves conducting several denial-of-service attacks between the infected devices and a web server. The tests are divided into two scenarios. In the first scenario, the detection of a denial-of-service attack is validated using federated learning with the Flower framework. Uninfected Raspberry Pi devices are used for the distributed processing and real-time classification of anomalous traffic. The identification of attacks is detailed in Figure 12.

In the second scenario, we validate the integrated solution, where attacks are identified using the results obtained in scenario one and the hosts receive rules from the HIDPS controller to suppress anomalous traffic. The reaction times to suppress the attacks are measured, generating graphs, tables and forms of the obtained results. The HIDPS solution is detailed in Figure 12.

### 4.1. Implementation Structure

In addition to the botnet, elements of the proposed security solution were included. Each of the components used is described below.

- Three intelligent devices installed on a Raspberry Pi 3 and using the Linux Raspbian distribution as an operational system. On the tests to be executed, the intelligent devices are infected with Mirai and receive orders from the Mirai controller to attack a web server that is installed on the local network. The intelligent devices start to exhibit bot behavior due to the Mirai network. These devices also receive the HIDPS software, which is responsible for mitigating attacks.
- A web server, installed on a personal computer with a Core i5 7500 processor, 8 GB of RAM and a Linux Elementary operational system, which is defined as our target.
- The Mirai controller, installed on a notebook with a I5 3360 processor, 8 GB of RAM and Ubuntu 16.04.
- The NIDS controller, responsible for monitoring network traffic, managing the federated learning server and registering new rules and updates from the hosts. Besides the IDS controller, we installed a DNS server on the same machine. The software was installed on a notebook with a I5 3360 processor, 8 GB of RAM and Ubuntu 16.04.
- A Layer 2 switch with port mirroring to interconnect all devices. All the elements were configured on the same VLAN.

**Figure 13:**
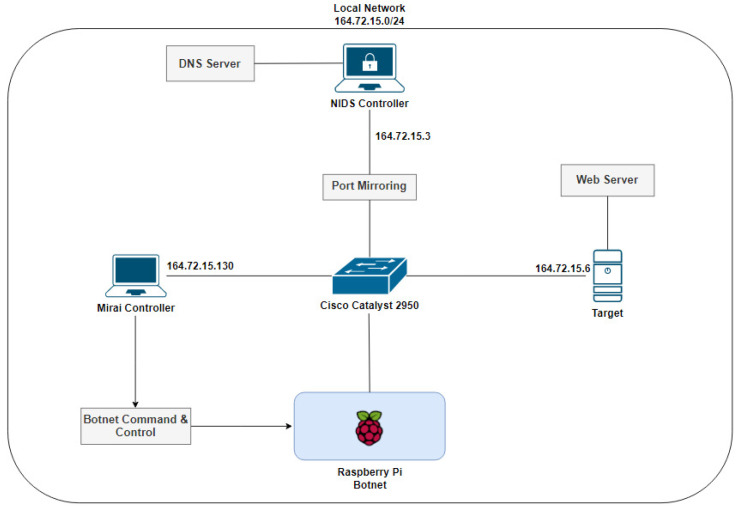
Real test scenario with NIDS and Mirai controller.

All devices were configured within the same network. A DNS server was installed on the NIDS controller to resolve the domain name from internal devices, required by the bots to resolve the Mirai controller’s address. The interconnection of the devices follows the topology shown in Figure 13.

Thus, the IoT devices installed on each Raspberry Pi 3’s board were ready to receive Python 3’s requests and reply packets from the IDPS and Mirai controllers. We then carried out a Mirai attack, on our private network, and transformed the IoT devices into bots on the Mirai botnet.

The bots received commands to execute HTTP flood attacks directed to the target. After the attack, Apache 2 was installed on the target device, with a default configuration as an HTTP server, without any improvement in its settings to alter the security or response time.

The HTTP flood attack had the following parameters:

- Target address;
- IP Port;
- Duration;
- Domain;
- Method.

In the NIDS controller, there is a federated learning server instance running in order to connect to Raspberry Pi 3 clients and apply the network traffic dataset training. The FL server uses network data captured during botnet attacks to classify each packet as normal or an anomaly. After classification and prediction, each client determines which rules need to be activated in order to mitigate the botnet.

To monitor the traffic generated by the bots, Wireshark was installed and used on the target. We configured Wireshark to run during all tests and capture all packets received at the target. The behavior of the packets sent from the infected bots to the target was analyzed. Wireshark was also used on the Mirai controller to capture packets between bots and the controller, to identify behaviors that would characterize an infected device being controlled by the botnet and to allow the creation of prevention rules to block the malicious traffic on the IDS.

As described in [10], distributed denial-of-service attacks could result in the target becoming unavailable, by raising the number of TCP connections that the server can respond to or by generating a large amount of traffic, overloading the servers along the rest of the network to the point they will not be able to respond to legitimate requests anymore.

Thus, the primary indicator analyzed in our tests was the transfer rate in bits, within a one-second interval received by the target. The transfer rate was obtained through data collected on Wireshark, monitoring the interface that received all the HTTP connections directed to the web server. In this way, all the received packets from the HTTP server could be visualized with Wireshark, generated by three bots within the network.

We defined test scenarios referring to detection and mitigation, where the behavior of the Mirai HTTP flood attack was observed through detection in the NIDS structure and mitigated through the HIDPS structure after rule activation. In the following scenarios, we defined rules on the HIDPS controller to interrupt the connection between the bots and the target after its activation through each federated learning client after the training, evaluation and classification procedure. A detailed description of each scenario follows in the subsequent subsections.

In this work, we chose to set up an environment with a real botnet, and this brought some difficulties in comparing the efficiency of our proposal with other published works. We compare the accuracy of this solution with two other works, highlighting the differences between them and our solution in the Results section in terms of the NIDS solution.

### 4.2. Test Scenarios

The testing segment is divided into two parts: the first one is focused on the training and detection step through the NIDS architecture, and the second one is focused on the HIDPS activity in order to mitigate attacks directly in malicious smart devices. At the end of the NIDS procedure, rules are activated through the HIDPS database to apply mitigation.

#### 4.2.1. Detection through NIDS

The test scenario uses the federated learning structure containing one central server and three smart devices acting as training clients. During this procedure, a prediction and classification step is performed to detect new input-captured data. Each training step is analyzed in terms of accuracy and loss to present the proposed model’s efficiency when differentiating between anomalies and benign network data. Then, it is compared to other related works that utilize NIDS with deep learning (either centralized or federated) in attack scenarios to validate the proposed model.

The base dataset, used to determine each client’s knowledge during the federated learning procedure, is obtained through a detection module. New input data are generated by the same module but without a static classification. This dataset is used in a real scenario with the Mirai botnet in order to study and obtain results in a near-real-time attack and detection procedure. This dataset contains 15 features with flow and signature data to be trained during the federated learning stage, with the main goal being to analyze and classify new input data as they are being captured in the detection module.

The federated learning method is initialized through the server and it sends new input data to all clients through sockets. The structure, containing all training settings, is defined hereafter:

- Three rounds of 1D-CNN;
- All clients train the model with 20 epochs in each round;
- The batch size is 32;
- The dataset is split into 80% for training and 20% for testing;
- Communication between server and clients is achieved by sockets.

The scenario consists of three infected smart devices applying a HTTP flood attack to the target’s web server. The NIDS structure performs the detection in order to obtain the necessary rule to activate DDoS mitigation through the HIDPS. The test with detection and mitigation is performed three times in order to obtain the following results:

- Aggregated accuracy, aggregated loss and execution time in the last training round by the fault-tolerant federated averaging algorithm;
- Rule activation through the HIDPS after DDoS detection by federated clients;
- HTTP flood attack mitigation directly in the smart device;
- Target and smart device availability through Wireshark.

With the three infected smart devices, an HTTP flood attack was performed on the target. During the attack, the NIDS was activated to apply federated learning, training, evaluation and new data detection. To check the training and detection performance, the accuracy, loss and execution time results were obtained in terms of training and evaluation through the fault-tolerant federated averaging aggregation algorithm.

Thus, during an attack, the federated learning structure trains a pre-obtained model serving as a knowledge base and then classifies each new packet that is captured in near-real time. Each client uses deep learning to train the model and applies detection after learning each pattern’s behavior.

At the end of FL execution, the rule is activated through the federated clients in such a way that the infected smart devices consume it through Kafka. The rule activation works through a Kafka producer, while the infected smart devices act as Kafka consumers in order to be mitigated.

#### 4.2.2. Mitigation through HIDPS

This scenario focuses on mitigation by providing the end devices with rules to be executed based on the type of attack detected by the NIDS. After the NIDS detects the type of attack and informs the HIDPS orchestrator, it sends an order to the IoT devices to execute the mitigation rule. The goal of this scenario is to observe the whole solution and measure the mean time from the identification to the mitigation of an attack.

The test scenario monitored the behavior of a DDoS attack orchestrated by Mirai in our test network. Three bots composed the botnet, being controlled by the Mirai controller, executing HTTP flood attacks aiming to overwhelm the server. The following parameters were defined to execute the attack:

- Attack type: HTTP flood;
- Target IP: 172.16.15.6;
- Elapsed time: 200 s.

**Figure 14:**
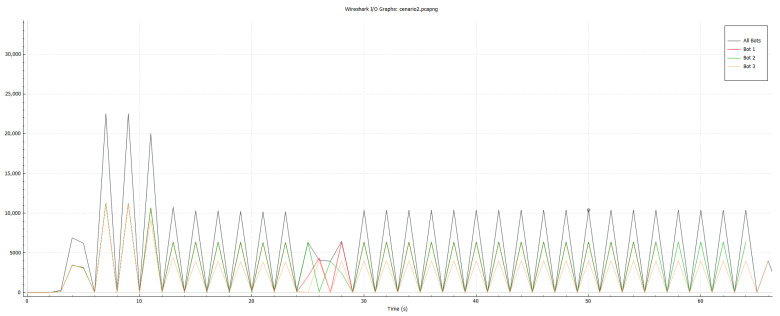
HIDPS results in terms of bytes × seconds during real-time mitigation procedure.

The duration of the attack was defined as 200 s, and from this moment, captured packets were received from the target server using Wireshark. The result is shown in Figure 14, detaching the total rate sent by the server from the rate received by each bot. It was observed that the maximum rate was 3 Mbits/s. Each bot was responsible for, at most, 1 Mbits/s, which represents a linear function between the number of bots and the total traffic.

In the tests performed, an attack was initialized, following the parameters described above; however, between 64 and 65 s of the attack, the NIDS registered a new rule on the HIDPS controller, requesting that any traffic sent to the target by the bots was blocked by the IPTables installed on the bot.

## 5. Results

Considering the NIDS and HIDPS scenario, particularly with its integration through the fog orchestrator using the mitigation structure referenced in Section 3, the two scenarios presented in Section 4 were executed in order to obtain detection and mitigation results with a real botnet performing DDoS attacks.

In this section, the proposed model’s results are divided into the following entities: the NIDS and HIDPS execution results and the benchmarking of NIDS- and HIDPS-related works.

### 5.1. NIDS Execution Results

The results below are derived from the proposed scenario to validate the denial-of-service detection capability described in Section 4.2.1. In each one of the three testing scenarios, three Raspberry Pi devices acted as federated learning clients in order to train the captured model through Suricata and Spark; then, the mitigation rule was applied to stop each infected device.

Three types of results were obtained: accuracy, to show the training and testing efficiency; loss, to show each client’s learning rate; and the execution time. These metrics are used to evaluate the performance of the anomaly-based detection model, with its methodology described in Section 4 and Section 4.2.1, in order to establish its strong performance when compared to works such as [32,33].

**Figure 15:**
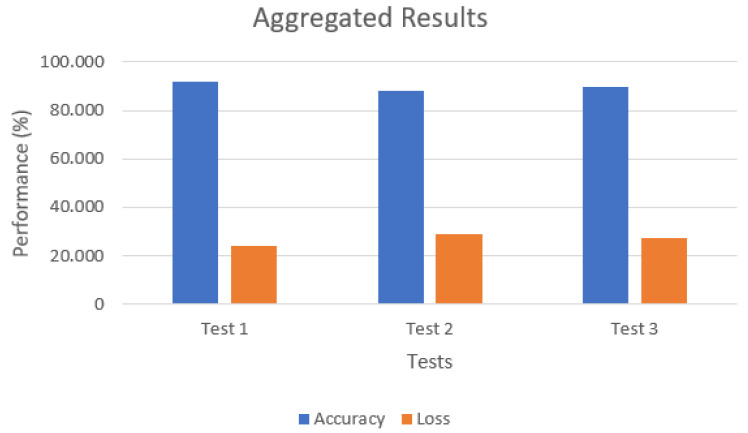
Aggregated federated learning results through FedAvg.

All scenarios’ results are shown in Table 4. Table 5 shows the mean square of each parameter. These results are analyzed and compared to those of related works in the next subsection. Figure 15 shows the aggregated results in a graphic.

With the NIDS, we can conclude that the general accuracy reached almost 90% during training and detection, with less than 30% loss. This loss value indicates that the detection architecture shows a great learning rate, thus being able to detect and classify new input in a more profound manner. The federated learning execution time is approximately five minutes when training the local dataset generated through Suricata and Apache Spark.

Considering its decentralized structure with low-end microarchitecture devices, a low bandwidth and ML capabilities, 90% accuracy in a near-real-time scenario with a real botnet is impressive when compared with centralized scenarios containing high-end devices. The NIDS execution time obtained in all three testing phases is low considering the context of a massive DDoS attack occurring simultaneously, and considering the fact that a deep learning algorithm is deployed at each low-end device to train, test and detect malicious data.

Another observation regards security and privacy issues in the detection phase. Different from a centralized scenario, the FL scenario considers a diversity of endpoints and reduces failure points during detection. With more endpoints in a Secure Sockets Layer environment, the training, testing and detection procedure offers greater privacy than other related works, such as [32,33].

### 5.2. HIDPS Execution Results

After each FL client exits the detection phase, the mitigation structure initiates the final phase through the HIDPS, as described in Section 4.2.2, in order to stop attacks from infected devices. The metrics used to evaluate the HIDPS performance were the time that each rule was executed and the rule-consuming time for each malicious device.

The results show an immediate decrease in traffic from the infected devices, which indicates that the rule was applied successfully. An analysis of the time taken to apply new rules to devices was performed in the sequence.

First of all, each rule has its own application time. In our test scenario, the rule was an IpTables command to block traffic, and we measured the average time taken to execute this rule. For this scenario, the tests were repeated three times, obtaining the values given in Table 6.

As shown in Table 6, the standard deviation for the run time rule was nearly zero, considering *Y* as the average time for rule execution of 0.125 s.

Therefore, the reaction time in this scenario can be expressed by

(1) F(x)=x+0.125,

where *x* represents the time that the bot requires to consume the rule published on the Kafka topic, and *F*(*x*) is the time that each bot requires to converge.

To measure the mean time that a bot requires to consume the rules published on the Kafka topic, an average was calculated for the time taken to consume messages in Kafka by the bots. We used 10 values to compute the average and obtained the results given in Table 7. It is important to note that these results are specific to the constraints of this test scenario, as the value can vary depending on the number of messages published on the topic. A complete analysis of the Kafka consumption time is beyond the scope of this work.

From Equation (1) and the average time presented for the consumption of messages in Kafka in this scenario, we can conclude that the total time taken to receive and execute the rule is, on average, below 1 s.

Thus, from each time calculated during the rule execution and consumption phase, which was below 1 s considering its deviation, the detection and mitigation procedure lasts less than 6 min on average. In terms of mitigation, each time is considerably small and indicates the ability to stop DDoS attacks after their detection.

### 5.3. NIDS-Related Works: Benchmarking

This project is compared to three related works. The first one [32] involves the implementation of a NIDS with 1D-CNN in imbalanced and balanced datasets to obtain result metrics. The second work [33] implements a NIDS and HIDPS architecture in a cloud Computing environment with K-nearest neighbor (KNN) and neural network (NN) algorithms to perform training and detection. The third work, called FedACNN [34], consists of an edge-assisted IoT with an Intelligent Intrusion Detection System implemented through federated learning and deep learning using a CNN.

#### 5.3.1. Comparing NIDS-Related Works

In order to compare our results with the related works mentioned above, all proposed solutions, domains and test scenarios are presented and compared.

The authors in [32] propose an LSTM with 1D-CNN scenario to train and test an imbalanced dataset with 100 different features. The second work [33] proposes a HIDPS and NIDS integrated with a Cloud-IDS using a hybrid KNN and NN algorithm to apply anomaly detection in an NSL-KDD training dataset. Our work proposes a NIDS and HIDPS architecture with federated learning and the 1D-CNN algorithm to apply anomaly detection in a dataset built with real Mirai traffic. The third work [34] proposes an IDS with horizontal FL and edge computing using a CNN in each edge device, applying an NSL-KDD dataset to perform the training and classification. Instead of using FedAvg, this work proposes an algorithm called FedACNN, which aggregates the average models of clients and also the weighted model parameters.

Table 8 presents the differences between all works, and it shows the difference in the test scenarios between all works as well. The comparison considers the configuration parameters, attack methods, dataset parameters, etc.

These comparisons were made in order to establish the differences between the related works and our proposed model in terms of the solutions, structures, algorithms used, ML parameters and scenarios. Before comparing the obtained results during anomaly-based attack detection, all works are compared regarding their infrastructures, technologies and testing methods.

Thus, after describing the main differences between all works, the results in terms of training and detection accuracy are compared in order to reveal their differences in terms of efficiency and performance in detecting anomalies in the network.

#### 5.3.2. Azizjon’s 1D-CNN Work

**Figure 16:**
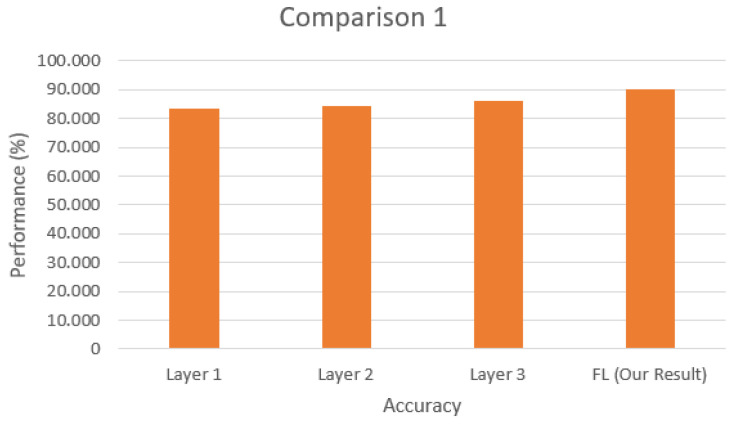
Comparing accuracy results between this project and Azizjon’s work.

The first work [32] obtains accuracy results using one to three layers of 1D-CNN with a learning rate of 0.0001, as in this project. In this project, we utilized a pre-obtained non-balanced model with 14 features, so the comparison was made with the imbalanced dataset’s (with 22 features) results obtained in the related work. Table 9 shows the accuracy values for all layers, and Figure 16 presents the comparison with this project.

The comparison made in Figure 16 shows the difference in training accuracy performance between the related work and this project. It is observed that the training and classification of traffic using the proposed hybrid architecture and federated learning is better than the performance of all layers in the related work. This indicates that, in a concatenated distributed environment with a mitigation architecture, the detection performance is superior, presenting operational advantages.

#### 5.3.3. Ghosh’s Hybrid NIDS and HIDPS Work

**Figure 17:**
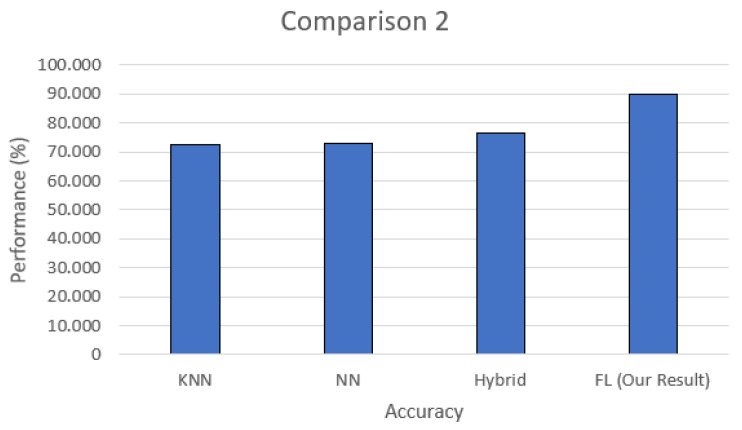
Comparing accuracy results between this project and Ghosh’s work.

The second work [33] combines a NIDS and HIDPS as well, but with a cloud Computing environment, implementing detection measures with KNN, NN and hybrid methods in a large flow of data packets. This work presents results in terms of accuracy, as shown in Table 10. Using the aggregation accuracy result, the comparison is realized in Figure 17, showing this project’s superiority in terms of training and detection.

The comparison made in Figure 17 shows the superiority of the detection and training using the federated learning model on an imbalanced dataset when compared to all methods used in the related work. The work applies KNN and NN individually, as well as using both in a NIDS and HIDPS architecture. This project, also using a hybrid architecture with distributed training, obtains better accuracy results from aggregation algorithms with multiple training clients.

These related works involve NIDS and even HIDPS detection through centralized machine learning methods using deep learning and neural networks. It was found that this project presents higher accuracy during training and detection in a distributed structure with federated learning, which naturally reduces the efficiency in order to increase privacy.

### 5.4. Dapeng’s FedACNN in Edge-Assisted IoT

**Figure 18:**
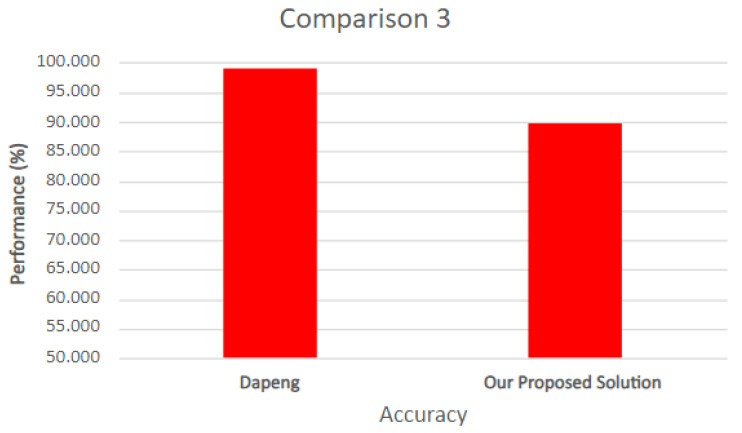
Comparing accuracy results between this project and Dapeng’s work.

The third work [34] applies a NIDS in an IoT infrastructure with edge computing using deep learning and federated learning to classify and identify anomalous data through the NSL-KDD dataset, performing edge device results’ aggregation through an algorithm called FedACNN. This work presents results in terms of accuracy, as shown in Table 11, containing all different epochs applied in the testing scenario. Using the aggregation accuracy result, the comparison is realized in Figure 18, showing the performance differences between these projects.

The comparison made in Figure 18 uses only the accuracy obtained with 20 epochs, because our work only used 20 epochs during FL training execution. It shows that Dapeng’s work achieves better accuracy regarding DoS detection, with a difference of 9%. Despite having better accuracy, our proposed solution shows great efficiency considering that it applies a botnet in a real scenario with a HIDPS integrated in order to apply mitigation in near-real time. Dapeng’s work uses NSL-KDD in a simulated scenario using only NIDS with a new aggregation algorithm, thus increasing the efficiency during FL training, but it does not apply mitigation as well.

Our project shows lower accuracy, but it has an anomaly detection and mitigation structure, thus increasing the security and privacy, and its application scenario is a real IoT with a fog computing infrastructure. Although Dapeng’s work has greater efficiency in terms of classification, it does not show greater privacy and security in an IoT decentralized structure, as with our proposed solution.

### 5.5. HIDPS-Related Works: Benchmarking

Based on the results obtained during the mitigation of DDoS attacks by the HIDPS in the proposed architecture, comparisons are made between this project and other related works that have implemented intrusion detection solutions on hosts. The focus of the comparison is to differentiate between network and infrastructure proposals of solutions in terms of configuration parameters (runtime and DDoS attack method).

#### 5.5.1. HIDPS Works’ Proposed Solutions

The work used as a point of comparison is a Hybrid Intrusion Detection and Prevention System (HIDPS) that combines an Anomaly-Based Intrusion Detection System (HIDPS) and Signature-Based Intrusion Detection System (SIDS) in a hybrid implementation [30]. In this study, a real IoT environment is utilized, and an ML dataset called Bot-IoT is applied, consisting of both legitimate and real anomaly traffic. The primary objective is to utilize the HIDPS with AIDS to detect zero-day attacks and SIDS to differentiate new attacks from well-known ones. The AIDS method employs a decision tree classifier in a centralized infrastructure to determine whether a test sample is malware or benign traffic. The SIDS is responsible for storing previously identified attacks from the AIDS system in its database, maintaining a history of attacks and malware.

Consequently, the signature-based structure with a decision tree classifier in the SIDS stage serves as a training method in the AIDS stage with the utilization of the one-class SVM algorithm on unknown traffic (traffic not detected with signature rules). This hybrid approach provides an ensemble to detect known attacks and predict new ones, thereby expanding the rule database over time.

Our project shares a similar objective in terms of combining anomaly-based and signature-based methods, but there are several differences. Firstly, our solution not only incorporates a HIDPS but also utilizes an ML approach with a Network-Based Intrusion Detection System (NIDS) to perform anomaly detection. Additionally, our project introduces a mitigation and prevention method in near-real time to effectively counter DDoS attacks using fog computing. Thus, our project combines two IDS approaches using decentralized ML with deep learning, instead of a decision tree classifier and one-class SVM, and it incorporates a mitigation component, resulting in a comprehensive prevention system.

Table 12 shows the differences between our project and the related work cited above.

#### 5.5.2. HIDPS Works Test Scenario

Table 13 shows the comparison between the two works in terms of the test scenarios used to validate each project and the results obtained. Our work emphasizes the reaction and mitigation time during the DDoS prevention stage to validate the HIDPS structure, and the related work only shows the ML results in terms of the confusion matrix, accuracy and true and false positive and negative rates. Therefore, there was no prevention and reaction time included in the related work, which was justified by the fact that it was not an IDPS.

## 6. Conclusions

This work focused on creating and deploying a cutting-edge hybrid Intrusion Detection System (IDS) that effectively merges Network-Based Intrusion Detection (NID) with Host-Based Intrusion Detection (HIDP). This integrated system, known as an Intrusion Response System (IRS), was designed to proactively identify and counter zero-day attacks. To accomplish this, we employed a decentralized federated learning system that leverages the power of deep learning for anomaly detection.

By utilizing this advanced approach, we were able to enhance the system’s ability to recognize and respond to unknown threats. Furthermore, we integrated attack mitigation capabilities into the system by leveraging a fog computing orchestrator. This enabled us to create and deploy custom rules for the mitigation of attacks, enhancing the overall security of the system.

The proposed model, which is a botnet detection and mitigation model for IoT networks, is robust in identifying and mitigating denial-of-service attacks, stopping attacks at the source. The federated learning method allowed the scalable use of a distributed computing model, where, to increase the processing capacity, it is sufficient to add more nodes, without wasting previous investments in fog computing nodes.

In comparison to our NIDS solution, three other works [32,33,34] utilized centralized machine learning, and the latter used federated learning with deep learning methods for anomaly detection in simulated scenarios. However, our work employed a decentralized learning technique in a real scenario involving real-time DDoS attacks. As a result, our approach presented superior performance in terms of accuracy, achieving an average accuracy rate of 89.753% during both training and attack detection. Notably, our solution also prioritized privacy concerns by leveraging distributed machine learning and a fog computing structure integrated with a HIDPS in a real-world setting.

In terms of results, the average accuracy, average loss and average execution time obtained during the anomaly detection procedure through FL were 89.753%, 26.705% and 303.904 s, respectively. Compared with [32], we achieved a 4% gain in terms of accuracy, and compared with [33], we achieved over 10% higher accuracy versus all deep learning methods used in the related work, and we only 9% lower accuracy compared with [34] when applying 20 epochs.

This superiority in terms of efficiency is determined by the utilization of federated learning with 1D-CNN, resulting in a decentralized training structure that increases privacy and reduces the training overload. The utilization of a federated training and detection procedure through deep learning minimizes the impact of a single point of failure (SPOF) and reduces the workload of each device because of the ML decentralization. Thus, the superior accuracy is due to the fact that multiple devices share training and detection results in a real scenario with a balanced dataset, applying 1D-CNN to detect each piece of malicious data.

As a future goal, we aim to explore this model within a software-defined networking (SDN) framework, incorporating OpenFlow-enabled switches into the scenario devised for this study. This endeavor will enable us to quantify the mitigation time in a real equipment environment. Furthermore, we plan to extend the application of this model to virtual machines in data centers, thereby introducing an additional layer of security. Additionally, as a continuation of this research, we intend to integrate this solution with cloud orchestrators, facilitating seamless integration and management in cloud computing environments.

## Figures and Tables

**Table 1:** Comparing HIDPS-related works and test scenarios.

Technology	IoT	HIDS	NIDS	Federated Learning	Fog Computing
Advantages	Enhanced automation and efficiency, optimization of processes and resources	Provides detailed visibility at the host level	Network-wide visibility and detection, monitors network traffic in real time	Preserves data privacy and security, enables collaborative learning without data sharing	Low latency and improved response times
Disadvantages	Security and privacy concerns, zero-day vulnerabilities	Limited coverage for network-based attacks, increased overhead on the host	Performance impact on network, false positives and negatives	Communication and synchronization overhead	Limited scalability, complexity in managing distributed resources
Application Scenarios	Smart homes and cities, environmental monitoring	Detection of file system anomalies, compliance monitoring	Intrusion detection in network traffic, network anomaly detection	Collaborative predictive modeling, mobile and edge device applications	Real-time data processing at the edge

**Table 2:** List of rules and their contexts.

Context	Rule
Network	Port scan detection
	DDoS attack detection
	DNS attack detection
Resource	Processes with excessive memory use
	Processes with excessive processing usage
	Processes with excessive usage of HD memory
	High temperature
Known vulnerabilities	Default passwords
	Standard open ports
	Configuration files in standard and unprotected directories

**Table 3:** Project’s dataset feature columns.

Feature	Description
flow_id	Integer type, referring to flow identification.
src_ip	String type, referring to the source IP address of the network packet.
dest_ip	String type, referring to the destination IP address of the network packet.
src_port	Integer type, referring to the source port of the packet.
dest_port	Integer type, referring to the destination port of the packet.
proto	String type, referring to the application protocol that the packet is associated with.
severity	Integer type, referring to the severity level of the captured event.
hour	Integer type, referring to the capture hour of the event.
minute	Integer type, referring to the capture minute of the event.
seconds	Integer type, referring to the capture second of the event.
pkts_to_server	Integer type, referring to the number of packets sent to the destination server.
pkts_to_client	Integer type, referring to the number of packets sent to the client.
bytes_to_server	Integer type, referring to the number of bytes sent to the destination server.
bytes_to_client	Integer type, referring to the number of bytes sent to the client.

**Table 4:** Federated learning results obtained during anomaly-based detection procedure through deep learning.

Test	Accuracy (%)	Loss (%)	Time (s)
1	91.647	23.974	297.098
2	88.125	28.978	304.633
3	89.488	27.165	309.982

**Table 5:** Final anomaly detection from NIDS results.

Average Accuracy (%)	Average Loss (%)	Average Time (s)
89.753	26.705	303.904

**Table 6:** Time to execute the mitigation rule in each infected device.

Test Suites	Average Runtime Rule (s)	Standard Deviation (s)
3	0.125	0.001

**Table 7:** Time to consume mitigation rule from Kafka in each infected device.

Test Suites	Average Runtime Rule (s)	Standard Deviation (s)
10	0.546	0.002

**Table 8:** Comparing NIDS-related works.

Work	[32]	[33]	[34]	This Proposed Model
Detection System	NIDS	HIDPS, NIDS, Cloud-IDS	NIDS	HIDPS, NIDS (IRS)
ML Structure	Centralized	Centralized	Federated Learning	Federated Learning
Deep Learning Algorithm	LSTM, 1D-CNN	KNN, NN	CNN	1D-CNN
Attack Methods	DoS, Backdoor	DoS, Probe, U2R, R2L	DoS, Probe, U2R, R2L	DDoS (HTTP Flood)
Dataset	UNSW NB15	NSL-KDD	NSL-KDD	Proposed Model’s Dataset
Dataset Parameters	22 used features	25 used features	Not indicated	15 used features
Training Parameters	32 batch size, 64 filter size, 200 epochs (centralized)	Not indicated (information gain)	128 batch size, 10 nodes, 5 rounds, 10 to 40 epochs	32 filter size, 3 nodes, 3 rounds, 20 epochs (per client)
Scenario	Simulated Scenario	Simulated Scenario	Simulated Scenario	Real Scenario

**Table 9:** The 1D-CNN results with “imbalanced” UNSW NB15 data in Azizjon’s work.

Method	Accuracy (%)
1	83.190
2	84.130
3	85.860

**Table 10:** Cloud NIDS and HIDPS accuracy results obtained in Ghosh’s work.

Methods	Accuracy (%)
KNN	72.490
NN	73.030
Hybrid	76.540

**Table 11:** FedACNN accuracy results with 10 to 40 epochs obtained in Dapeng’s work.

Epochs	Accuracy (%)
10	98.730
20	99.020
40	99.120

**Table 12:** Comparing HIDPS-related works’ proposed solutions.

Work	[30]	This Proposed Model
HIDPS Goals	Detection	Detection and Prevention
Attack Type	DDoS, Key Logging	DDoS (HTTP Flood)
Technologies	IoT Gateway, Machine Learning	Fog Computing, IoT Gateway
Scenario	Simulated Scenario	Real Scenario

**Table 13:** Comparing HIDPS-related works’ test scenarios.

Work	[30]	This Proposed Model
Test Scenario	Simulated IoT network with DDoS attack dataset	Real IoT network with botnet
Results Obtained	Machine learning output	Elapsed time and average runtime rule (prevention time)

## Data Availability

The data presented in this study are available on request from the corresponding author. The data are not publicly available due to using traffic in an isolated and real scenario with a real botnet, containing unavailable information with ethical restrictions about the network used in this project.

## References

[1] Kotha H.D., Gupta V.M. (2018). IoT application: A survey. Int. J. Eng. Technol..

[2] Chunka C., Banerjee S., Gupta D.S. (2023). A secure communication using multifactor authentication and key agreement techniques in internet of medical things for COVID-19 patients. Concurr. Comput. Pract. Exp..

[3] Silva S.S., Silva R.M., Pinto R.C., Salles R.M. (2013). Botnets: A survey. Comput. Netw..

[4] Bertino E., Islam N. (2017). Botnets and Internet of Things Security. Computer.

[5] Zoppi T., Ceccarelli A., Bondavalli A. (2021). Unsupervised Algorithms to Detect Zero-Day Attacks: Strategy and Application. IEEE Access.

[6] Dutra B.V., Martins L.M.C.E. (2019). HIDS by signature for embedded devices in IoT networks. Proceedings of the Actas de las V Jornadas Nacionales de Ciberseguridad.

[7] Ahmad Z., Shahid Khan A., Wai Shiang C., Abdullah J., Ahmad F. (2021). Network intrusion detection system: A systematic study of machine learning and deep learning approaches. Trans. Emerg. Telecommun. Technol..

[8] Liu H., Lang B. (2019). Machine learning and deep learning methods for intrusion detection systems: A survey. Appl. Sci..

[9] da Mata R., Filho F., Mendonca F., Fares A., de Sousa Junior R. Hybrid Architecture for Intrusion Prevention and Detection in IoT Networks. Proceedings of the 2021 Workshop on Communication Networks and Power Systems (WCNPS).

[10] Schiller C.A., Binkley J., Harley D., Evron G., Bradley T., Willems C., Cross M. (2007). Botnet: The Killer Web App.

[11] Vargas Martinez C., Vogel-Heuser B. (2021). A Host Intrusion Detection System architecture for embedded industrial devices. J. Frankl. Inst..

[12] De Carvalho Bertoli G., Pereira Júnior L.A., Saotome O., Dos Santos A.L., Verri F.A.N., Marcondes C.A.C., Barbieri S., Rodrigues M.S., Parente De Oliveira J.M. (2021). An End-to-End Framework for Machine Learning-Based Network Intrusion Detection System. IEEE Access.

[13] Phan T.V., Bauschert T. (2022). DeepAir: Deep Reinforcement Learning for Adaptive Intrusion Response in Software-Defined Networks. IEEE Trans. Netw. Serv. Manag..

[14] Modi A.S. Review Article on Deep Learning Approaches. Proceedings of the 2018 Second International Conference on Intelligent Computing and Control Systems (ICICCS).

[15] Lauzon F.Q. An introduction to deep learning. Proceedings of the 2012 11th International Conference on Information Science, Signal Processing and their Applications (ISSPA).

[16] O’Shea K., Nash R. (2015). An Introduction to Convolutional Neural Networks. arXiv.

[17] Samat N.A., Salleh M., Ali H. (2020). The Comparison of Pooling Functions in Convolutional Neural Network for Sentiment Analysis Task. Recent Advances on Soft Computing and Data Mining: Proceedings of the Fourth International Conference on Soft Computing and Data Mining (SCDM 2020), Melaka, Malaysia, 22–23 January 2020.

[18] Li T., Sahu A.K., Talwalkar A., Smith V. (2020). Federated Learning: Challenges, Methods, and Future Directions. IEEE Signal Process. Mag..

[19] Sharma I., Sharma A., Gupta S.K. Asynchronous and Synchronous Federated Learning-based UAVs. Proceedings of the 2023 Third International Symposium on Instrumentation, Control, Artificial Intelligence, and Robotics (ICA-SYMP).

[20] Shaheen M., Farooq M.S., Umer T., Kim B.S. (2022). Applications of Federated Learning; Taxonomy, Challenges, and Research Trends. Electronics.

[21] Gupta A., Gupta D.S. (2022). A survey on green unmanned aerial vehicles-based fog computing: Challenges and future perspective. Trans. Emerg. Telecommun. Technol..

[22] Alzahrani R.J., Alzahrani A. (2023). A Novel Multi Algorithm Approach to Identify Network Anomalies in the IoT Using Fog Computing and a Model to Distinguish between IoT and Non-IoT Devices. J. Sens. Actuator Netw..

[23] von Sperling T.L., de Caldas Filho F.L., de Sousa Júnior R.T., e Martins L.M.C., Rocha R.L. Tracking intruders in IoT networks by means of DNS traffic analysis. Proceedings of the 2017 Workshop on Communication Networks and Power Systems (WCNPS).

[24] Kasinathan P., Costamagna G., Khaleel H., Pastrone C., Spirito M.A. DEMO: An IDS Framework for Internet of Things Empowered by 6LoWPAN. Proceedings of the 2013 ACM SIGSAC Conference on Computer & Communications Security.

[25] da Silva Cardoso A.M., Lopes R.F., Teles A.S., Magalhães F.B.V. Real-Time DDoS Detection Based on Complex Event Processing for IoT. Proceedings of the 2018 IEEE/ACM Third International Conference on Internet-of-Things Design and Implementation (IoTDI).

[26] Jun C., Chi C. Design of Complex Event-Processing IDS in Internet of Things. Proceedings of the 2014 Sixth International Conference on Measuring Technology and Mechatronics Automation.

[27] Hodo E., Bellekens X., Hamilton A., Dubouilh P.L., Iorkyase E., Tachtatzis C., Atkinson R. Threat analysis of IoT networks using artificial neural network intrusion detection system. Proceedings of the 2016 International Symposium on Networks, Computers and Communications (ISNCC).

[28] Pacheco J., Hariri S. IoT Security Framework for Smart Cyber Infrastructures. Proceedings of the 2016 IEEE 1st International Workshops on Foundations and Applications of Self* Systems (FAS*W).

[29] Sabahi F., Movaghar A. Intrusion Detection: A Survey. Proceedings of the 2008 Third International Conference on Systems and Networks Communications.

[30] Khraisat A., Gondal I., Vamplew P., Kamruzzaman J., Alazab A. (2019). A Novel Ensemble of Hybrid Intrusion Detection System for Detecting Internet of Things Attacks. Electronics.

[31] Fotiadou K., Velivassaki T.H., Voulkidis A., Skias D., Tsekeridou S., Zahariadis T. (2021). Network Traffic Anomaly Detection via Deep Learning. Information.

[32] Azizjon M., Jumabek A., Kim W. 1D CNN based network intrusion detection with normalization on imbalanced data. Proceedings of the 2020 International Conference on Artificial Intelligence in Information and Communication (ICAIIC).

[33] Ghosh P., Mandal A., Kumar R. (2015). An Efficient Cloud Network Intrusion Detection System. Adv. Intell. Syst. Comput..

[34] Man D., Zeng F., Yang W., Yu M., Lv J., Wang Y. (2021). Intelligent Intrusion Detection Based on Federated Learning for Edge-Assisted Internet of Things. Secur. Commun. Netw..

[35] Nguyen T., Marchal S., Miettinen M., Fereidooni H., Asokan N., Sadeghi A.R. DÏoT: A Federated Self-learning Anomaly Detection System for IoT. Proceedings of the 2019 IEEE 39th International Conference on Distributed Computing Systems (ICDCS).

[36] Flower Documentation. https://flower.dev/docs.

